# Disparities in Thyroid Screening and Medication Use in Quebec, Canada

**DOI:** 10.1089/heq.2018.0051

**Published:** 2019-07-11

**Authors:** Kathrin Stoll

**Affiliations:** Department of Family Practice, Faculty of Medicine, University of British Columbia, Vancouver, Canada.

**Keywords:** thyroid, screening, medication use, population data, Canada, disparities

## Abstract

**Background:** No studies have examined the frequency of thyroid screening in the Canadian population, and whether thyroid screening and medication use vary by sex, race, income, and preexisting health conditions.

**Methods:** Using data from the 2011, 2012 cycles of the Canadian Community Health Survey, we report rates of thyroid screening among Quebec residents ≥35 (*n*=7024) and rates of thyroid medication use among Quebec residents ≥35 (*n*=16,081). We examine variations in medication use and screening by sex, age, race, immigration status, access to a regular doctor, and health conditions that have been linked to thyroid disease.

**Results:** Of the Quebec residents ≥35, 10.3% reported taking thyroid medication and 0.4% reported that the last blood test a physician ordered was to check for a new thyroid condition. Canadian-born residents and those who identified as White reported higher medication use and screening rates, compared to immigrants and those who identified as visible minorities. Racial disparities were especially pronounced, with White Quebec residents reporting three times greater odds of thyroid screening than visible minorities. The strongest predictors of both thyroid medication use and screening were access to a regular doctor. Despite women being eight times more likely to suffer from thyroid disease, women were not significantly more likely to be screened, compared to men (odds ratio=1.38, 95% confidence interval: 0.74–2.60).

**Discussion:** Strategies are needed to decrease disparities in thyroid screening and medication use. Interventions that target health systems (e.g., increasing physician supply), providers (continuing professional education modules about thyroid disease for family physicians), and recipients of care (multilanguage public awareness campaigns and posters at walk-in clinics that describe common symptoms of different thyroid disorders) should be implemented and tested.

## Introduction

Thyroid disease is one of the most underdiagnosed health conditions in the world; it is estimated that 50–60% of people affected by thyroid disease in North America are not aware of their condition.^1,2^ Early diagnosis is important, to improve quality of life and to prevent progression of the disease and other health conditions that are linked to untreated thyroid disorders.

Hypothyroidism is the most common of the thyroid dysfunctions and the most common hormone deficiency.^3^ Consequences of untreated hypothyroidism include increased vascular resistance, hypertension and increased cardiovascular mortality, impaired intellectual function, depression, and memory loss, especially in the elderly. In women of childbearing age, hypothyroidism has been linked to irregular menstruation, impaired neurological development of the fetus, anovulation and impaired fertility, higher rates of spontaneous abortion, reduced fetal growth, and developmental abnormalities. Untreated Hyperthyroidism (either primary or treatment induced) is associated with tachycardia, hypertension, atrial fibrillation, peripheral edema, angina, heart failure, corneal damage, double vision, optic nerve compression, vision loss, and osteopenia.^4^

Routine screening for thyroid dysfunction is not recommended in people without symptoms, but is indicated in patients with nonspecific symptoms and the following risk factors: personal history of thyroid disease, strong family history of thyroid disease, diagnosis of autoimmune disease, past history of neck irradiation, drug therapies such as lithium, men over age 60, women over age 50, and women with a history of pregnancy loss, preterm delivery, or infertility.^5^

It is estimated that 1 in 10 Canadians suffer from thyroid disease.^1^ Women are at increased risk, with one in eight women affected by thyroid disorder during their lifetime.^2^ Although a family history of thyroid disease increases the likelihood of developing the disease among offspring, the causes of thyroid disorders are mostly unknown. Most thyroid disorders are chronic and can be managed with medication.^2^

The majority of published studies about thyroid disease in Canada examine the prevalence and treatment of thyroid cancer. *Very little is known about thyroid disorders among Canadian residents and there have been no published reports of thyroid screening rates that are based on a representative sample.* In addition, it is unclear whether screening rates resemble medication use rates among at-risk populations and vary by socioeconomic status, race, gender, and access to a regular primary care physician. Given the estimate that 50% or more cases of thyroid disease are missed, it is timely to undertake a population study of thyroid screening and medication use.

## Methods

### Study population

We undertook an analysis of the 2011/2012 Canadian Community Health Survey (CCHS), a cross-sectional survey of Canadian residents 12 years of age and older. Each year ∼65,000 residents are interviewed about their health status and health behaviors. Respondents can complete the interview in French, English, or a wide range of other languages. Participation is voluntary, and the survey excludes persons living on reserves in the provinces; full-time members of the Canadian Forces; the institutionalized population; and persons living in the Quebec health regions of Nunavik and Terres-Cries-de-la-Baie-James. In addition, Statistics Canada provides survey weights for each participant corresponding to the number of Canadian residents who are represented by the respondent. The survey weights ensure that estimates derived from survey data are representative of the Canadian population. Data from the CCHS and other Statistics Canada surveys are available for download and analysis, to researchers at Canadian postsecondary institutions. Ethics approval for analysis of this data is not required because the data are publicly available, that is, made available through government legislation/regulation and as a result appropriately protected by law.

### Rationale for analysis

As there are no published studies about thyroid screening, based on a Canadian population, it is important to provide an empirical reference point for the thyroid screening analysis presented in this article. The medication use analysis provides such a framework. Specifically, by knowing which groups are more likely to use thyroid medication, we know which groups are more likely to need medication and thus should be prioritized for screening.

### Outcome variables

#### Thyroid screening

**FIG. 1.**
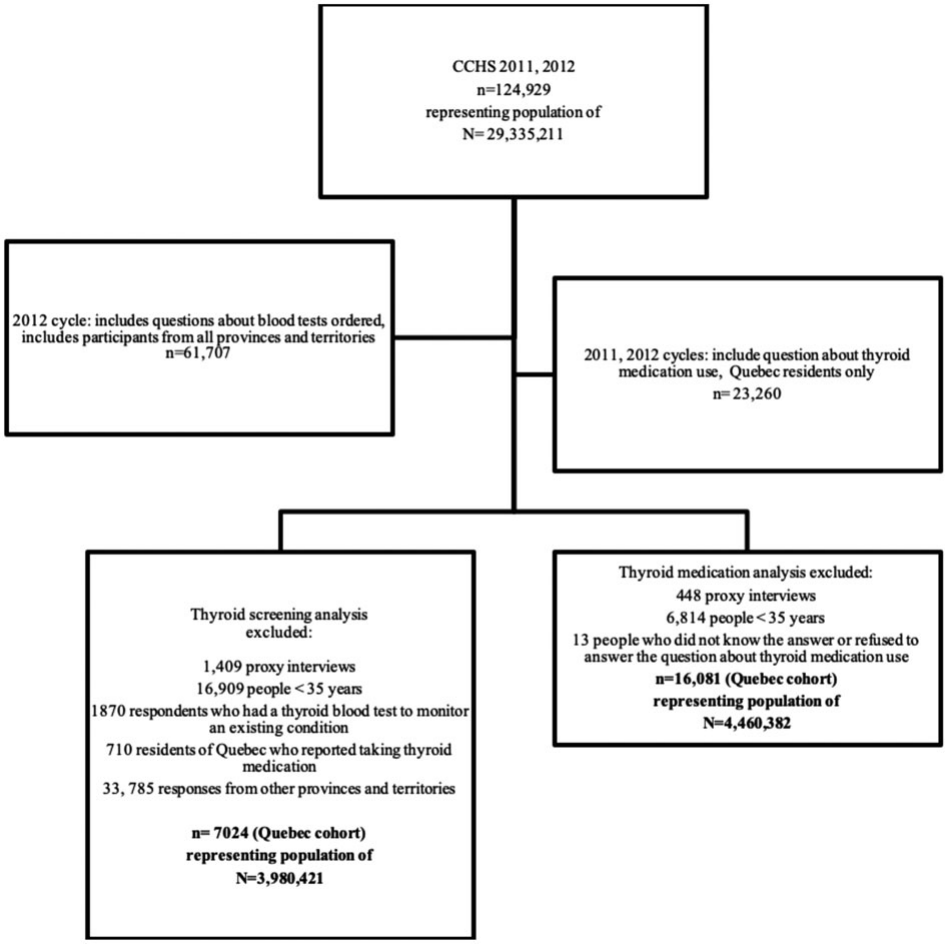
Sample size flow chart. CCHS, Canadian Community Health Survey.

The 2012 cycle of the CCHS included a section about blood tests. All respondents aged 35 or over were asked whether a health professional ordered a blood test in the past 12 months. People who answered yes to this question were asked why the most recent blood test was ordered. Response options were as follows: (1) for assessment as part of a general physical check-up; (2) to monitor an existing health condition; (3) to check for a new specific disease or health condition; (4) as the result of an emergency (e.g., heart attack, food poisoning, and car accident); and (5) other-specify. Next, respondents were asked for which health condition the last blood test was ordered. Response options were: (1) high cholesterol or other heart-related condition; (2) diabetes; (3) thyroid; (4) prostate; (5) infectious disease; (6) liver function; (7) hormone related; and (8) other. The numerator for the current analysis included the number of people ≥35 who reported that the last blood test that was ordered for them was to investigate a new thyroid condition. The denominator included all people who were at risk for a new thyroid condition and excluded residents of Quebec who were already on thyroid medication, those who received a blood test to monitor an existing thyroid condition, and respondents <35 years. The denominator included people who did not have any blood work done during the 12 months preceding the interview. The purpose of this analysis was to determine what percentage of Quebec residents received a blood test to investigate a new thyroid condition (Fig. 1). Because these rates only apply to the last blood test ordered, they will be lower than screening rates over a 12-month period, but nevertheless give important insight about thyroid screening in Quebec.

#### Thyroid medication use

We also examined thyroid medication use in a representative provincial sample, using 2 years of data (2011, 2012). Questions about medication use were only asked of residents of Quebec (*In the past month, that is, from 1 month ago to yesterday, did you take: thyroid medication such as Synthroid or Levothyroxine*?). The numerator included residents of Quebec ≥35 who reported taking thyroid medication. The denominator included all residents of Quebec ≥35. Because the likelihood of being diagnosed with a thyroid disorder increases with age and questions about thyroid screening were only asked of respondents ≥35 in the CCHS, we excluded respondents under 35 years of age. We also excluded data from proxy interviews to improve data accuracy. Proxy interviews were interviews conducted with another family member.

### Statistical analysis

We report frequencies and proportions of thyroid medication use and screening, by subpopulations at increased risk of thyroid disorder and by selected sociodemographic characteristics (i.e., visibility minority and immigration status). All reported proportions have been weighted, that is, we used the sampling weights provided by Statistics Canada to derive reliable estimates. Thyroid screening and medication use rates were stratified by the following variables: Sex, age, country of origin, race, income, access to a regular doctor, and the following physician-diagnosed disorders that have lasted at least 6 months: mood disorders (such as depression, bipolar disorder, mania, or dysthymia), anxiety disorders, diabetes, and bowel disorders (such as irritable bowel syndrome, colitis, or Crohn's disease). These chronic health conditions are linked to undiagnosed thyroid disease or are more likely to affect people with thyroid dysfunction. The list of variables was developed in consultation with an experienced endocrinologist and contingent on questions asked in the 2011, 2012 cycles of the CCHS.

Associations between variables are expressed as odds ratios (ORs) and confidence intervals (95% CI). ORs and CIs were calculated, using logistic regression analysis. When calculating adjusted ORs (AORs) the variables were entered as follows into the hierarchical logistic regression model: Block 1: sex, age, income categories, race, and country of origin; Block 2: access to a regular doctor; and Block 3: diagnoses of mood disorder, anxiety disorder, diabetes, and bowel disorder. Sample weights for this analysis were averaged, and the original sampling weights were divided by the mean weight, to avoid erroneous estimates of statistical significance. SPSS 22 was used for all analyses.

## Results

### Thyroid medication use

The prevalence of thyroid medication use among residents of Quebec was 7.5% (all ages); the prevalence among residents ≥35 was 10.3%. Women had nearly four times the odds of taking thyroid medication compared to men. Canadian born and White respondents had increased odds of taking medication compared to foreign born residents and those from visible minorities (Table 1). Residents who reported incomes in the lowest deciles had twice the odds of taking medication for a thyroid disorder compared to residents in the highest income quintile. The odds of Quebec residents with a regular doctor being on thyroid medication were nearly six times greater than the odds of residents without a regular doctor. Medication use rates were similar among people with and without a mood disorder, but were significantly higher among people with an anxiety disorder. Quebec residents who were diagnosed with diabetes or a bowel disorder had nearly two times greater odds of being on thyroid medication compared to residents who were not diagnosed with these conditions (Table 1).

**Table 1.** Frequencies and Weighted Proportions (%) and Odds and Weighted 95% Confidence Intervals of Thyroid Screening (*n*=7024) and Thyroid Medication Use (*n*=16,081), for Sociodemographic and Other Indicators

	Thyroid medication use, *n* (%)	Thyroid medication use, OR (95% CI)	Thyroid screening, *n* (%)	Thyroid screening, OR (95% CI)
Quebec residents	1994 (10.3)		36 (0.4)	
Gender				
Female	1590 (15.6)	3.79 (3.36–4.28)	27 (0.5)	1.38 (0.74–2.60)
Male	404 (4.7)	1.00	9 (0.4)	1.00
Age				
≥50	1777 (13.4)	3.02 (2.65–3.45)	21 (0.4)	1.04 (0.54–1.98)
<50	217 (4.9)	1.00	15 (0.4)	1.00
Country of origin				
Canada	1803 (10.7)	1.32 (1.13–1.54)	33 (0.5)	1.66 (0.57–4.82)
Other	104 (8.3)	1.00	3 (0.3)	1.00
Race				
White	1841 (10.6)	1.37 (1.13–1.66)	34 (0.5)	3.21 (0.63–16.27)
Visible minority	57 (8.0)	1.00	2 (0.2)	1.00
Income^a^				
Deciles 1 and 2	624 (12.9)	2.01 (1.67–2.42)	9 (0.3)	0.68 (0.27–1.96)
Deciles 3 and 4	532 (12.3)	1.91 (1.59–2.30)	8 (0.8)	1.63 (0.66–4.00)
Deciles 5 and 6	393 (10.3)	1.56 (1.29–1.89)	7 (0.3)	0.54 (0.18–1.67)
Deciles 7 and 8	265 (8.1)	1.19 (0.98–1.46)	5 (0.3)	0.55 (0.17–1.74)
Deciles 9 and 10	180 (6.9)	1.00	7 (0.5)	1.00
Has regular doctor?				
Yes	1894 (12.2)	5.71 (4.49–7.26)	34 (0.5)	7.47 (1.30–42.95)
No	99 (2.4)	1.00	2 (0.1)	1.00
Mood disorder				
Yes	156 (11.6)	1.14 (0.93–1.41)	8 (1.0)	2.43 (0.94–6.30)
No	1835 (10.3)	1.00	27 (0.4)	1.00
Anxiety disorder				
Yes	190 (15.3)	1.62 (1.35–1.96)	11 (1.7)	4.81 (2.21–10.48)
No	1802 (10.0)	1.00	24 (0.4)	1.00
Diabetes				
Yes, type 1	5 (12.3)	1.95 (1.68–2.26)	0 (0)	2.47 (1.49–4.10)
Yes, type 2	231 (17.1)		3 (1.4)	
No	1568 (6.9)	1.00	33 (0.3)	1.00
Bowel disorder				
Yes	106 (21.1)	1.92 (1.56–2.37)	2 (0.3)	0.78 (0.13–4.63)
No	1476 (15.3)	1.00	34 (0.4)	1.00

^a^ This variable originally had 10 categories based on the adjusted ratio of respondents' total household income to the low-income cutoff corresponding to their household and community size. It provides, for each respondent, a relative measure of their household income to the household incomes of all other respondents in the same province. The Territories are excluded from this derived variable.

CI, confidence interval; OR, odds ratio.

When all variables were entered into the model, the odds of being on thyroid medication were highest for Quebec residents with a regular doctor (AOR=3.90, 95% CI: 3.04–5.02), women (AOR=3.28, 95% CI: 2.89–3.72) residents who were 50 years or older (AOR=2.42, 95% CI: 2.10–2.80), those in the lowest income category (AOR=1.61, 95% CI: 1.32–1.96), and residents diagnosed with diabetes (AOR=1.56, 95% CI: 1.33–1.84) or a bowel disorder (AOR=1.53, 95% CI: 1.22–1.92). The odds of being on thyroid medication were not significantly greater for White residents (AOR=0.99, 95% CI: 0.76–1.27) or those who were born in Canada (AOR=1.11, 95% CI: 0.90–1.38). This adjusted analysis is based on a sample size of 15,233.

### Screening for a new thyroid condition

Of 7024 residents, 36 (0.4%) reported that the last test a physician ordered for them was a blood test to check for a new thyroid condition. The odds of being screened were 1.3 times greater among women than men and similar between age groups. However, Canadian born residents and those who identified as White reported higher screening rates compared to immigrants and refugees and those who identified as visible minorities. Racial differences were especially pronounced, with White Quebec residents reporting three times greater odds of thyroid screening than visible minorities. Screening rates varied by income deciles, but not in a systematic pattern. Respondents without a regular medical doctor had very low screening rates (0.1%). The odds of being screened for a new thyroid condition were seven times greater if the respondent reported having a regular doctor.

Thyroid screening was more commonly reported among respondents with chronic diseases. The odds of being screened were about two to three times greater among people who had been diagnosed with a mood disorder or diabetes. Residents of Quebec with a diagnosed anxiety disorder had almost five times greater odds of reporting a thyroid blood test, but residents with a bowel disorder had reduced odds of being screened (Table 1).

AORs could not be calculated for the outcome, because only 36 residents were screened for a new thyroid condition. For each independent variable there should be no fewer than 10 observations or events in the binary outcome variable, with the less common outcome (i.e., the likelihood of being screened) determining the maximum number of independent variables that can be included in the model.^6^

### Disparities between the odds of medication use and the odds of screening

Comparison of the odds of being screened versus the odds of being on medication has the potential to identify groups who are not adequately screened but likely to need medication.

When examining differences in ORs between medication use and screening rates, the odds of taking thyroid medication were higher than the odds of being screened for the following groups: women (OR=3.79 for medication use vs. 1.38 for screening), residents ≥50 years of age (OR=3.03 vs. 1.04), and residents in the lowest two income deciles (OR=2.01 vs. 0.68).

## Discussion

Our findings indicate that 7.5% of Quebec residents (all ages) and 10.3% of residents 35 or older took thyroid medication in 2011/2012. Access to a regular doctor was associated with seven times greater odds of being screened for thyroid disease and five times greater odds of being on thyroid medication. Others have documented increased screening rates among Canadians with access to a regular physician.^7,8^ While major depression does not appear to increase the incidence of thyroid disease,^9^ hyper-and hypothyroidism are described as causes of mood disorders in the *Diagnostic and Statistical Manual of Mental Disorders (Fourth Edition)*.^10^ In addition, symptoms of mood disorders are similar to symptoms of some thyroid conditions; thus, it is advisable to screen patients with diagnosed mood disorders or symptoms of mood disorders for thyroid conditions. Our findings support this practice, as people with a mood disorder were significantly more likely to get screened compared to those without mood disorders. The high rates of screening among residents with mood and anxiety disorders compared to the lower rates of thyroid medication use among these groups likely indicate that physicians order thyroid screening tests, to rule out physiological causes of common symptoms of mood and anxiety disorders.

## Health Equity Implications

Thyroid medication use was significantly lower among visible minorities and immigrants to Canada. Similar disparities were observed in screening rates, that is, White residents were thrice more likely to be screened compared to residents who identified with a visible minority. These findings add to a growing literature that documents disparities in screening for minorities and new immigrants in North America.^11,12^ Interventions to reduce these inequities have been evaluated and implemented for colorectal cancer screening,^11^ but not for thyroid disease screening. Unmet health care needs among Canadian immigrants have been linked to language barriers, problems with obtaining information about how to access and navigate health services, financial barriers and cultural differences on part of patients, and lack of cultural competency among health care providers.^13^ While these barriers might impact on peoples' ability to find a regular doctor, the problem of critical physician shortages in Canada remains and affects people's access to diagnostic tests and secondary (i.e., specialist) care.

Given that thyroid disease is eight times more prevalent in women compared to men,^14^ it was surprising to see that the odds of being on thyroid medication for women ≥35 years (compared to men ≥35) were only 3.79. The odds of screening for women over 35 were also low (OR=1.38). Low thyroid screening rates for women should be investigated further, to determine possible reasons. Are women less likely to share symptoms with their primary care physicians because they attribute symptoms of thyroid disease to other factors? Do primary care physicians miss opportunities for thyroid screening of women for the same or other reasons? Do the findings indicate systemic inequities? Gender inequities in the provision of health care have been documented, even after controlling for gender differences in care seeking and preferences for treatment. In one study, women in the United Kingdom who experienced hip pain were significantly less likely (compared to men) to have been referred to a specialist or to have consulted with an orthopedic surgeon.^15^

The large gap between the odds of being screened for a thyroid disorder versus the odds of being on medication for Canadian residents 50 years of age and Canadian residents with very low incomes indicates that older and poor Canadians might not be screened appropriately.

In Canada, a country with universal, publicly funded health care, patients should in theory be able to access care, regardless of their sex, race, age, or socioeconomic status. However, Fowler et al. demonstrated gender and age inequities in ICU admissions and length of stay at Ontario hospitals among men and women, controlling for illness severity and other confounders.^16^ Women over age 50 who were admitted to the ICU had a greater risk of dying within 1 year of admission, compared to their male counterparts.^16^ Despite hundreds of studies documenting health disparities in Canada, little has been done to address the issue, especially with respect to racial disparities.^17^

To bridge this gap, Anderman^18^ offers a clear description of how health care providers can reduce health inequities in Canada. For example, physicians are encouraged to ask patients about social challenges in a nonthreatening manner, take extra time to understand patients' complex health and social needs, help vulnerable patients access services and resources, provide culturally safe care, and enlist the help of patient navigators, if available. Ongoing training and support for health care providers in how to address social determinants of health in clinical practice is an important part of the solution, as are collaborations with community groups who serve vulnerable populations.

One cost-effective strategy to increase screening and detection of thyroid disease is to distribute posters (in several languages) at walk-in clinics (i.e., clinics often visited by people without a regular doctor) across the country that inform patients about the symptoms of thyroid disorder and encourage them to discuss these symptoms with the physician. Some posters could be directed at higher risk groups, such as women over age 50 and people who suffer from autoimmune disorders. Educational modules directed at family doctors are another strategy to increase awareness among physicians about thyroid disease and indications for screening.

Although controversial, private pay health services are emerging around Canada, to give Canadian residents with the means to pay out of pocket the option of expedited access to health services (especially diagnostic tests). In Ontario, the most populous of Canadian provinces, residents can choose one of five thyroid blood profiles and pay for blood tests privately, without having to visit a physician.^19^ Thyroid blood profiles appear to be among the most commonly requested blood tests as information about these tests feature prominently on the company website. While companies that offer private blood tests increase access to such services, it is contingent on the ability to pay and will further exacerbate inequities in health care access in Canada.

### Limitations

CCHS respondents were only asked about the last blood test ordered; thus, we could not determine how many respondents were screened annually. We could not assess thyroid screening or medication use among other high-risk groups, such as postpartum women and those with a family history of the disease, because questions about these conditions were not included in the CCHS. In addition, the CCHS likely did not capture every Quebec resident who received thyroid screening as some participants might not have known that their doctor ordered the test.

Finally, the number of Quebec residents who reported a thyroid screening test for a new condition was very low (i.e., 36 cases). For this reason it is important to replicate the analysis in a larger sample, ideally including questions about thyroid screening over a 1-year period and questions that allow for exclusion of all people with existing thyroid disease. With a larger sample size it would also be possible to examine screening rates for Canadian residents who belong to more than one group (e.g., women over age 50, residents from a visible minority who do not have a regular doctor, and immigrants who also report very low incomes), to assess inequities in thyroid screening associated with intersecting characteristics/risk factors.

## Conclusion

We found variations in screening and medication use rates that have important implications for public health. Future research ought to focus on replicating the screening analysis in a larger representative sample. Interventions that target health systems (e.g., training more family physicians and developing an up-to-date database of family physicians who take new patients), providers (educational modules about thyroid disease for family physicians and modules about social determinants of health), and recipients of care (public awareness campaigns and posters that describe common symptoms of different thyroid disorders) should be implemented and tested.

## Abbreviations Used

CCHS: Canadian Community Health Survey
CI: confidence interval
OR: odds ratio
